# The Roles of Envelope Glycoprotein M in the Life Cycle of Some Alphaherpesviruses

**DOI:** 10.3389/fmicb.2021.631523

**Published:** 2021-02-19

**Authors:** Chunmei Li, Mingshu Wang, Anchun Cheng, Renyong Jia, Qiao Yang, Ying Wu, Dekang Zhu, Xinxin Zhao, Shun Chen, Mafeng Liu, Shaqiu Zhang, Xumin Ou, Sai Mao, Qun Gao, Di Sun, Xingjian Wen, Bin Tian

**Affiliations:** ^1^Institute of Preventive Veterinary Medicine, Sichuan Agricultural University, Chengdu, China; ^2^Key Laboratory of Animal Disease and Human Health of Sichuan Province, Sichuan Agricultural University, Chengdu, China; ^3^Avian Disease Research Center, College of Veterinary Medicine, Sichuan Agricultural University, Chengdu, China

**Keywords:** alphaherpesviruses, glycoprotein M, entry, fusion, the viral life cycles

## Abstract

The envelope glycoprotein M (gM), a surface virion component conserved among alphaherpesviruses, is a multiple-transmembrane domain-containing glycoprotein with a complex N-linked oligosaccharide. The gM mediates a diverse range of functions during the viral life cycle. In this review, we summarize the biological features of gM, including its characterization and function in some specicial alphaherpesviruses. gM modulates the virus-induced membrane fusion during virus invasion, transports other proteins to the appropriate intracellular membranes for primary and secondary envelopment during virion assembly, and promotes egress of the virus. The gM can interact with various viral and cellular components, and the focus of recent research has also been on interactions related to gM. And we will discuss how gM participates in the life cycle of alphaherpesviruses.

## Introduction

Herpesviruses are a linear double-stranded DNA virus that infects various animals (Engel et al., 2015). Herpesviruses can be classified into three subfamilies, alpha-, beta-, and gamma-herpesviruses (Mettenleiter et al., 2006; Davison et al., 2009). All herpesviruses share a common virion structure from the outside to the inside: envelope, tegument, capsid, and core (Mettenleiter, 2002). Their genomes are composite of long and short unique regions (UL, US) and two inverted repeat sequences, internal repeat sequences, and terminal repeat sequences (Wu et al., 2012). And ICTV online (10th) report show that the alphaherpesviruses contain five recognized genera featuring 42 species, among them the virus that studies more thorough has herpes simplex virus 1 and 2 (HSV-1, HSV-2), bovine herpesvirus 1 (BHV-1), equine herpesvirus 1 and 4 (EHV-1, EHV-4), varicella-zoster virus (VZV), pseudorabies virus (PRV), Marek’s disease virus (MDV), infectious laryngotracheitis virus (ILTV), waterfowl duck enteritis virus (DEV; Sadaoka et al., 2010; Wu et al., 2012; Yang et al., 2014; Dhama et al., 2017). And we will introduce the functions of related gM around these important viruses. The alphaherpesviruses’ envelope is similar to the lipid bilayer structure, which plays a decisive role in connecting with the outside and stabilizing the inside (Weed and Nicola, 2017). The glycoprotein M (gM), located on the envelope, is highly conserved among the alphaherpesviruses and participates in multiple phases of the viral life cycle. During initiate entry and infection, gM participates in virus-induced cell fusion to release the nucleocapsid to the cytoplasm. In the middle and late stages of viral infection, gM is involved in the virus’s primary and secondary envelopment and the recruitment of other proteins to specific sites for assembly and the successful release of infectious progeny virions. Current studies on gM have found that the gM can interact with various viral and host cellular components, and there is a complex mechanism for regulating the virus replication cycle. Due to the limitations of the current research results about gM, in this review, we will discuss how gM participates in and regulates the life cycle of some alphaherpesviruses.

## The Characteristics of the gM Gene and its Encoding Protein

The genome of alphaherpesviruses contains multiple open reading frames (ORFs), each encoding a specific protein. The gM gene was encoded by different ORF in different viruses. For example, gM is encoded by the UL10 gene, including HSV-1 (McGeoch et al., 1988), HSV-2 (Dolan et al., 1998), PRV (Brack et al., 1999), BHV-1 (Wu et al., 1998), ILTV (Veits et al., 2003), MDV (Cai et al., 1999), and DEV (Wu et al., 2012). In other alphaherpesviruses, gM is encoded by ORF50, including VZV (Davison and Scott, 1986; Sadaoka et al., 2010) or ORF52, including EHV-1 (Telford et al., 1992) and EHV-4 (shown in Table 1). Although gM has homologous genes in alphaherpesviruses, suggesting that it is a common role at certain stages of the viral life cycle, its importance is different (Browne et al., 2004). For instance, gM is nonessential for viral growth in HSV-1 (Baines and Roizman, 1991), HSV-2, PRV (Dijkstra et al., 1996, 1997), BHV-1 (König et al., 2002), VZV (Yamagishi et al., 2008), EHV-1 (Osterrieder et al., 1996; Shakya et al., 2017), EHV-4 (Ziegler et al., 2005), and ILTV (Fuchs and Mettenleiter, 1999). However, gM is essential for the viral replication of MDV (Tischer et al., 2002).

**Figure 1:**
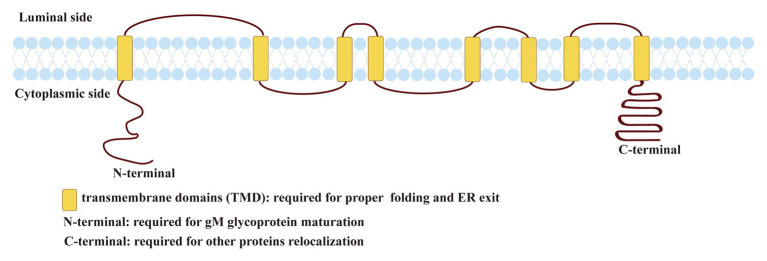
Predicted topology of herpes simplex virus 1 (HSV-1) glycoprotein M (gM). The topology of HSV-1 gM was predicted using the TMHMM transmembrane topology prediction server (http://www.cbs.dtu.dk/services/TMHMM/; Baines and Roizman, 1993; Striebinger et al., 2015; Striebinger et al., 2016).

**Table 1:** gM gene of specific alphaherpesviruses and its function.

Virus	Gene	Protein	Type of protein	Function in life cycle	References
HSV-1	UL10	gM	type III transmembrane protein	modulate the viral fusion machinery with gN	Striebinger et al., 2015
HSV-2	UL10	gM	/	/	Dolan et al., 1998
BHV-1	UL10	gM	type III transmembrane protein	Help gN and VP22 incorporate to virion	Lipińska et al., 2006; Pannhorst et al., 2018
EHV-1	ORF52	gM	type III transmembrane protein	involved in virus egress and fusion	Rudolph and Osterrieder, 2002
EHV-4	ORF52	gM	type III transmembrane protein	Ensure virus egress and cell-to-cell spread	Ziegler et al., 2005
VZV	ORF50	gM	type III transmembrane protein	Ensure virus cell-to-cell spread	Yamagishi et al., 2008; Sadaoka et al., 2010; Lebrun et al., 2018
PRV	UL10	gM	/	inhibits membrane fusion and involved in axonal sorting and transport	Brack et al., 1999; Kratchmarov et al., 2015
MDV	UL10	gM	/	essential for virus growth in cultured cells	Cai et al., 1999; Tischer et al., 2002
ILTV	UL10	gM	/	/	Veits et al., 2003
DEV	UL10	gM	/	/	Wu et al., 2012

**Figure 2:**
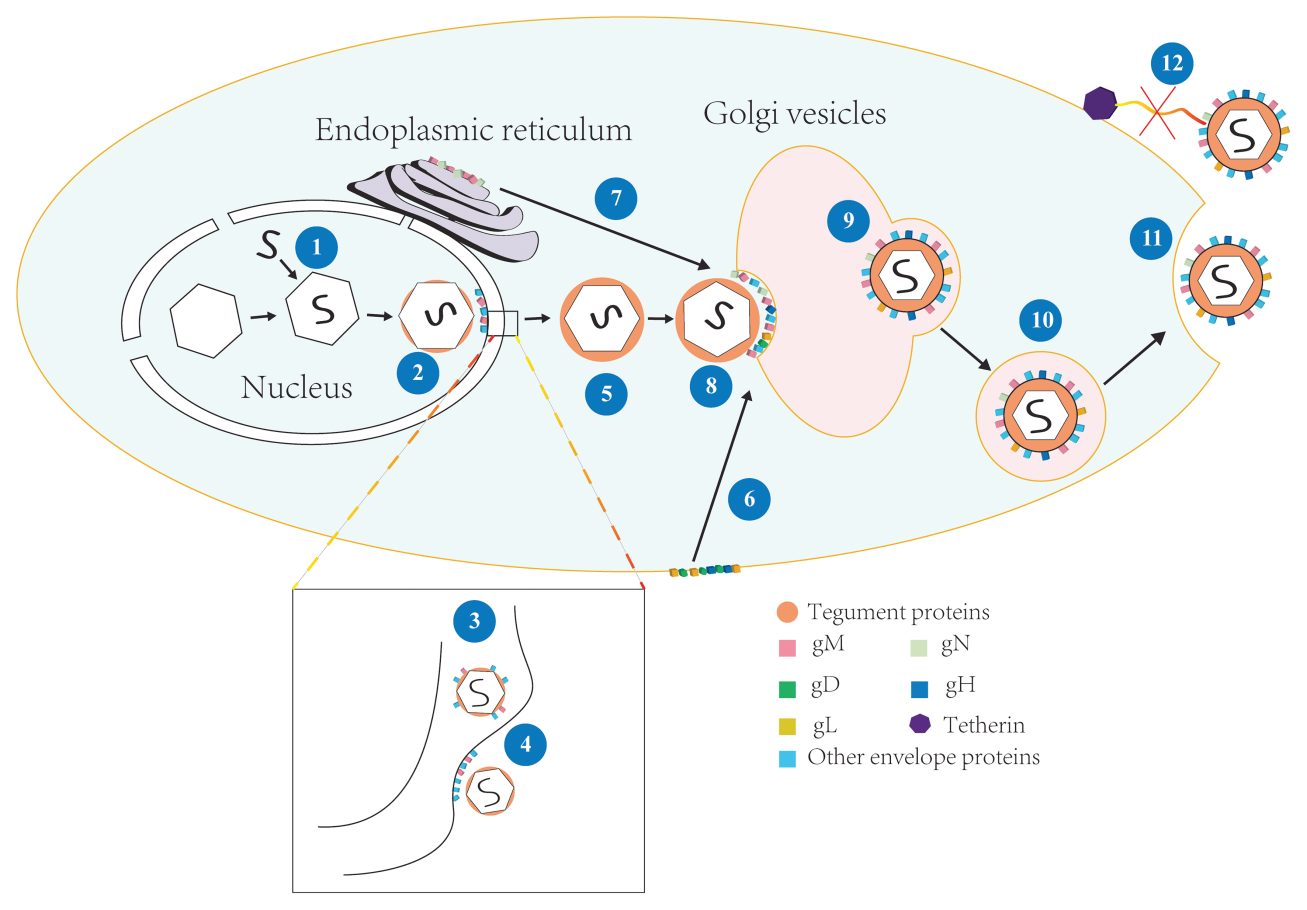
The involvement of gM in the life cycle of HSV-1. (1) Viral genome is assembled into the capsid to form the nucleocapsid (Skepper et al., 2001); (2) The nucleocapsid acquires a small number of tegument proteins in the nucleus (Baines et al., 2007; Wills et al., 2009); (3) Immature virions budding in the inner nuclear membrane and enter the perinuclear space, meanwhile, gM is assembled into the primary envelope (Baines et al., 2007; Zhang et al., 2009); (4) The primary envelope is de-envelopment at the outer membrane and the naked nucleocapsid is released into the cytoplasm; (5) The naked nucleocapsid acquires a large number of tegument proteins in the cytoplasm (Johnson and Baines, 2011; Maringer et al., 2012); (6) gD, gH, and gL located on the surface of cell membrane were internalized by gM and relocated to Golgi apparatus (Ren et al., 2012); (7) gN and gM located in the endoplasmic reticulum (ER) form complex and matures, and then transfer to Golgi apparatus (Graul et al., 2019); (8) The nucleocapsid is wrapped by tegument proteins and obtained the ultimate envelope at the Golgi apparatus (Turcotte et al., 2005); (9)–(10) The vesicles derive from Golgi apparatus and wrap the enveloped virions and transported them to the cell membrane (Sugimoto et al., 2008); (11) Progeny virions released by exocytosis at cell membrane (Turcotte et al., 2005; Johnson and Baines, 2011); and (12) gM antagonized the tetherin and virions were successfully released (Blasius et al., 2006).

Herpes simplex virus 1 gM is a type III transmembrane protein comprised of 473 amino acids encoded by the UL10 ORF, including 1,422 base pairs (shown in Figure 1). HSV-1 gM is predicted to contain eight membrane-spanning domains, and it is very tightly inserted into the membrane or envelope, so its stable structure plays an important role in antagonizing substances that can permeate lipid bilayer (Van Cleemput et al., 2020). gM can first express as a 47-kDa precursor that resides in the endoplasmic reticulum (ER). When it matures, it can be modified with a complex N-linked oligosaccharide to form glycosylated proteins with a size between 53 and 63 kDa (Baines and Roizman, 1991, 1993). All transmembrane domains of HSV-1 gM are required for its proper folding and ER exit (Striebinger et al., 2015). And the cytoplasmic tail domain contains several putative trafficking motifs, including dileucine motifs, endocytosis motifs, and tyrosine-based motifs, which can interact with clathrin adaptor complexes to transport cell surface proteins to the proper site (shown in Figure 1; Striebinger et al., 2015, 2016). Moreover, the N-terminal domain of gM mainly responsible for maturing (shown in Figure 1; Crump et al., 2004). A similar trafficking motif structure is also present in VZV and plays an important role in the virus’s pathogenesis (Zerboni et al., 2018).

## Effects of gM on the Life Cycle of Some Alphaherpesviruses

Different viral life cycle processes have some similarities and differences in alphaherpesviruses. The similarities are as follows: alphaherpesviruses undergo two forms of replication, a latent infection that often occurs in the nervous system for long-term residency and is difficult to detect, and when the latent viruses are reactivated, it will enter lytic replication (Heldwein and Krummenacher, 2008; Yang et al., 2019). In the lytic replication cycle, gene expression of alphaherpesviruses begins with immediate early genes, then early genes, followed by late genes (Everett et al., 2004; Roizman and Zhou, 2015; Cohen and Kobiler, 2016). The secondary envelopment location is either Golgi or early endosome or autophagosome (Henaff et al., 2012; Lv et al., 2019). And many viruses are no definitive conclusion (Albecka et al., 2016; Lebrun et al., 2018). But it is generally accepted that secondary envelopment sites are located in Golgi because various glycoprotein and most tegument proteins accumulate in Golgi (Turcotte et al., 2005; Sugimoto et al., 2008; Johnson and Baines, 2011).

Here, we will show some of the differences: how the virus enters the cell, the proteins needed to start the infection, and the way the virions are released. BoHV-1 employs an endocytosis mechanism and a low-pH endosomal pathway for effective cell entry (Pastenkos et al., 2018). HSV-1 mainly employs virus-induced membrane fusion, and gD can interact with cell receptors (Zhang et al., 2011; Li et al., 2017), but VZV is gE to connected with cell receptors (Olson et al., 1998; Li et al., 2007) because VZV does not have gD homolog gene (Cohen, 2010). PRV causes virus-induced membrane fusion by combining gp50 with cell receptors, and it also utilizes low-pH-mediated endocytosis to enter the cell (Miller et al., 2019). For virion release, most viruses of Alphaherpesviridae can release virions into the supernatant in cell culture; MDV is an exception (Harmache, 2014; Heidari and Delekta, 2017).

Latent infection can only detect transcripts and has too much mystery, so let us take HSV-1 as an example to introduce the lytic infection of alphaherpesviruses. HSV-1 enters the host cell by fusion, which happens between the viral envelope and cytomembrane by interacting with the virus’s surface glycoproteins *via* specific cell surface receptors (Hadigal and Shukla, 2013; Rogalin and Heldwein, 2015; Zhou et al., 2015). This fusion process is regulated by both the host cellular and the viral proteins (Klupp et al., 2000; El Kasmi et al., 2018; Saiz-Ros et al., 2019). After fusion, virions undergo uncoating, and the viral nucleocapsids are transported to the nucleus and anchor at nuclear pores for injected viral genetic material into the nucleoplasm. The viral genome is then replicated and expressed, and further assembly of progeny capsids form intranuclear DNA-filled capsids (You et al., 2017). Subsequently, the nascent nucleocapsids undergo primary envelopment at the inner nuclear membrane and de-envelopment at the outer nuclear membrane (Skepper et al., 2001; Mettenleiter et al., 2013). The unenveloped nucleocapsids enter the cytoplasm to obtain a large number of tegument proteins, then undergo secondary envelopment at the Golgi apparatus, which is widely considered to be the site of secondary envelopment (Hambleton et al., 2004; Johnson and Baines, 2011; Buckingham et al., 2016), and the enveloped mature virions are transported to the cytomembrane by vesicles derived from the Golgi membrane and released to the extracellular space by exocytosis (shown in Figure 2). And the gM participates in the regulation of multiple stages in this life cycle, such as the entry of viruses into the cell, the primary and secondary envelopment of viruses, the assembly of viral components, and the release of virions. Next, we will discuss the functions of gM in detail.

### gM Impacts on Viral Entry

Alphaherpesviruses enter cells, which is the first step of a successful infection (Tiwari et al., 2006). Viruses are attracted to the cell surface by non-specific electrostatic adsorption and specific adsorption. When certain viral envelope glycoproteins bind to the specific receptors on the cell surface, the process of viral infection has been initiated (Weed and Nicola, 2017). The specific receptors of gD include nectin-1, nectin-2, herpes virus entry mediator (HVEM), and 3-O-sulfated heparan sulfate (3-O-S-HS). Different cell types would mainly choose one of the receptors (Shukla et al., 1999; Connolly et al., 2011). And the presence of cell surface receptors determine viral affinity for cells (Hadigal and Shukla, 2013) and determine whether intracellular conditions support the viral activity. The most widely accepted model is that alphaherpesviruses enter the cell by fusion. In other words, membrane fusion is the first essential step for the entry of alphaherpesviruses into host cells (Harrison, 2015; White and Whittaker, 2016). We were known that HSV-1 gB, gD, gH/gL are the “core role” in virus-induced fusion (Sathiyamoorthy et al., 2017), gM is a modulator in this process and implies that gM is involved in virus entry into the cell (El Kasmi and Lippé, 2015). VZV lacks the gD homolog in its genome, gB serves as the main fusion function (Oliver et al., 2017), and gM can enhance syncytium formation (Sadaoka et al., 2010), indicating that gM and gB have a synergistic effect in function.

Glycoprotein M has two conformations of “immature” and “mature,” which may result in differences in efficiency and specificity on fusion (Atanasiu et al., 2016). Although the molecular mechanism of how gM regulates the virus-induced fusion process is unclear, gM certainly interacts with a variety of cellular proteins in this process (Changotra et al., 2016; Polpitiya Arachchige et al., 2018), and it may provide favorable conditions for the viruses to enter cells and generate more infectious progeny virions, as well as gain an advantage in multiple infections.

Glycoprotein M did not affect the expression of bovine respiratory syncytial virus F protein that induced syncytium formation in recombinant BHV-1 infected cells, but gM is involved in the inhibition function of F protein (König et al., 2002). Besides, studies have also demonstrated that gM can inhibit the envelope and cellular membrane fusion induced by gB and gD under transfection conditions (Klupp et al., 2000), suggesting that gM does not function by degrading proteins that promote fusion. EHV-1 gM can antagonize antimicrobial β-defensins to facilitate virions to invade new hosts through airborne transmission, and interestingly, β-defensins was more conducive to viral adsorption and infection after it was antagonized by gM (Van Cleemput et al., 2020). In other words, gM can completely reverse the function of β-defensins, and the underlying mechanism remains to be investigated. Altogether, these findings suggest that the fusion process regulated by gM may be related to other proteins (König et al., 2002; Kim et al., 2013), and also shows that highly specialized and precise strategies have developed during pathogen entry into the cells, which reflects the process of mutual struggle and common progress between host and virus (Crump et al., 2004; White et al., 2008; Gomes et al., 2018).

### Role of gM in Primary Envelopment and Secondary Envelopment

The membrane fusion process involves virus-induced fusion and involves the process of primary and secondary envelopment. The nascent proteins’ subcellular location depends on its trafficking motifs, indicating that the protein might contain corresponding functions. gM can locate various membrane structures (Crump et al., 2004; Baines et al., 2007; Graul et al., 2019). gM may be recruited by UL31 and UL34 or an unknown mechanism unrelated with rearrangement of Golgi apparatus to reside in inner nuclear membrane (Turcotte et al., 2005; Desai et al., 2008; Wills et al., 2009; Zhang et al., 2009), and it is clear that gM nuclear exit requires XPO6 at late times. Subsequently, gM was localized to the Golgi (Boruchowicz et al., 2020). gM leaving the nucleus may also have something to do with γ_1_34.5 (Wu et al., 2016; Boruchowicz et al., 2020). Because gM can use indirect interaction with viral protein γ134.5 (Wu et al., 2016), the latter can recruit protein kinase C to the nuclear membrane and rearrange it, and promote gM incorporation into the virion during the primary envelopment at the inner nuclear membrane (shown in Figure 2; Baines et al., 2007). It also implies that gM might interact directly or indirectly with the capsid surface.

Glycoprotein M can also be localized to the Golgi apparatus, and when gM is co-expressed with other glycoproteins, then their localization may be changed by gM and relocated to the correct assembly site that can facilitate proteins rightly assemble to virion during secondary envelopment (Crump et al., 2004). For instance, when HSV-1 gN was expressed alone, gN was positioned on the ER, and it was co-expressed with gM, gN was recruited by gM to the Golgi apparatus (shown in Figure 2; El Kasmi and Lippé, 2015; Striebinger et al., 2016). The deletion of gM alone has little effect on virus replication (Browne et al., 2004; Ren et al., 2012). However, when gM is deleted with some tegument proteins or envelope proteins, the process of secondary envelopment of the virus is seriously damaged so that the ability of the progeny virions to infect cells successfully was significantly reduced (Leege et al., 2009; Maringer et al., 2012; Ren et al., 2012), suggesting that involvement of gM in the primary and secondary envelopment was closely related to its network of interactions.

### gM Interacts With Other Viral Components and Assists Their Assembly

The interaction between the components of the virus promotes its survival in the cell. gM, as an important protein of the envelope, has a homologous compound in the whole herpesviruses, which can interact with other viral components to form an interaction network and aid in forming a complete virion structure. The gM/gN complex exists in many herpesviruses (e.g., HSV-1, PRV, and BHV-1) and can be connected by a covalent disulfide bond between cysteine residues and non-covalent bond (Mach et al., 2005; Sadaoka et al., 2010; Striebinger et al., 2016), in BoHV-1, the glycine zipper motifs within transmembrane helices of gM are required for the ER release and maturation of the gM/gN complex, and it also interacts with other cellular proteins (Graul et al., 2019), such as soluble N-ethylmaleimide-sensitive factor attachment protein receptors (v-SNARE) and vesicle-associated membrane protein 3 (VAMP3; Kawabata et al., 2014; Wang et al., 2017), which facilitate the transport protein complex between membrane-enclosed intracellular organelles, thanks to technological advances, weak or transient but specific interactions between gM and nearly 60 proteins can now be detected using a proximity-dependent method (Boruchowicz et al., 2020). Therefore, these interaction relationships not only facilitate gM/gN transport from the ER to the Golgi apparatus and incorporate into the virion through the secondary envelopment, but also integrate the cell components into the virion and assist the progeny virions in entering the cell (Jahn and Scheller, 2006; van den Bogaart et al., 2010). Some viral proteins also assist in the transport process, when gM is deleted, and gM/gN complex cannot form, gN can promote and assemble to correct site of virions by VP22 (Fuchs et al., 2002; Pannhorst et al., 2018). There is a noticeable fact that the content of gN in progeny virions is lower than normal (Sadaoka et al., 2010), indicating that gN incorporate into virion is mediated by multiple proteins, but gM plays a crucial role in the effective assembly of gN.

Endocytosis can take glycoproteins to reach assembling virions at different rates (Albecka et al., 2016). The expression of gM causes other viral envelope proteins that do not contain a transport signal sequence to redirect to the Golgi compartment (Zhang et al., 2009). gD and the gH/gL complex do not encode endocytosis motifs (Lau and Crump, 2015). When they interact directly or indirectly with gM, gM can efficiently internalize them from the plasma membrane and vesicles bearing viral glycoproteins to facilitate their transport (Roberts and Baines, 2010), which may also involve the assistance of gK/UL20 complex to gM (Kim et al., 2013). And gM can internalize itself (Albecka et al., 2016). These studies revealed a mechanism is that gD and gH/L could localize to intracellular virus assembly compartments by gM or other proteins associated with gM (shown in Figure 2; Ren et al., 2012; Lau and Crump, 2015). Also, gM promotes the correct assembly of UL20 into virions because they interact with each other, indicating that gM plays an important role in promoting tegument and envelope proteins assembly (Stylianou et al., 2009; Maringer et al., 2012). All of the above indicates that gM involves incorporating viral proteins and the cell-to-cell spread of infection.

### gM Modulates Viral Egress and Spread

As virions mature, they need to be released successfully to spread and infect more uninfected cells. For highly cell-associated viruses, such as VZV (Zerboni et al., 2018), MDV (Wang et al., 2011), syncytium formation is required for efficient cell-to-cell spread. Viruses can affect the release process in many ways to spread to neighboring cells. In the early stages of mixed infection, HSV-1 gM can interfere with gp160 (glycoprotein 160, is a precursor of HIV gp41 and gp120) processing and matures and influence viral replication of human immunodeficiency virus (HIV; Polpitiya Arachchige et al., 2018). gM guarantees the HSV-1 “nutrition” supply and safeguard the “foundation” of the virus particles in mixed infection. Mature virions formed are required to release into the extracellular space *via* exocytosis. However, the host cell would take action to prevent this process. An antiviral effector of host cells, tetherin, captures the nascent virions by form tethers between the viral envelope and cell membrane, thereby inhibiting viral release and spread. HSV-1 gM can internalize tetherin and inactivate its function (shown in Figure 2; Blondeau et al., 2013). Studies have shown that tetherin can increase type I of interferon (IFN-I; Blasius et al., 2006), so gM antagonist tetherin, whether it also affects the IFN-I and provides a favorable condition for viral replication, it needs further studies (König et al., 2002). gM for many virus replications is nonessential, but the single deletion of gM also influence the viral release and spread. For example, gM-null mutants can cause the content of gH/gL complex has decreased in progeny virions and result in progeny virions exist delay phenomenon in the process of entry into the cells.

Meanwhile, it reduces the diffusion speed in cell-to-cell spread (Ren et al., 2012). Even if only gM single amino acid site is mutated, it can cause viral defects in intercellular infection (Pannhorst et al., 2018). However, because of the redundancy of functions performed between viral proteins (Zenner et al., 2013; Liu et al., 2015), this function of gM may have different effects on different viruses (Blasius et al., 2006; Liu et al., 2015).

## Conclusion

The degree of understanding of alphaherpesviruses life cycle determines the effectiveness of their prevention and treatment strategies. gM, as a highly conservative envelope glycoprotein, plays a wide range of functions in the alphaherpesviruses life cycle, involving the process from the virus enter cells to the viral assembly and release. Most studies are still on the superficial level of the influence of gM on viruses, which gives us a preliminary understanding of the roles of gM. When gM/gN complex has been completed, gN has lost its ability to inhibit the transporter associated with antigen processing (TAP; Verweij et al., 2011). In other words, the method of using gN to stop TAP has failed, whether the virus uses other pathways to block the antigen transport process, and how does it activate other pathways, because gM takes gN away from ER and activates some signaling proteins, or does it activate some proteins because gM gets to ER and gM is recognized by the associated proteins at ER? The virus escapes immunity in a variety of ways. As we know, β-defensins are secreted by most leukocytes and epithelial cells, and they are effectors of the innate immune system with potent antiviral activity (Holly et al., 2017; Meade and O’Farrelly, 2018). When the virus invades the host respiratory tract, β-defensins can chemoattract a broad swath of immune cell types. Subsequently, the host would activate innate immunity and adaptive immunity (Yang et al., 1999; Soruri et al., 2007), but EHV-1 gM can completely reverse the function of β-defensins (Van Cleemput et al., 2020), it may be that the information β-defensins communicates to immune cell is wrong, or it loses the function and cannot send relevant information. Other researchers believe that the cationic β-defensins electrostatically bind to the viral envelope and/or anionic surface glycoproteins, leading to the local aggregation of the virion and increasing the chance of infection (Van Cleemput et al., 2020).

Since most of the reports are related to the interaction of gM, there may also be a problem with the proteins that interact proteins with gM that whether the E-Syt proteins and gN compete for gM, as they seemingly have opposite effects, whether a specific protein or cell factors control the process in a different period to determine the function of the gM, whether the combination of gN or E-Syt and gM is chronologic. About 60 transport-related proteins interact with gM (Boruchowicz et al., 2020), indicating that each small step in the life cycle involves many proteins’ interaction and coordination, the key step of viral infection, which can be the ideal antiviral target. The more detailed molecular mechanisms that cause these phenomena need further exploration, which gives us a deeper understanding of gM’s roles and has profound significance in our understanding of herpesviruses’ life cycle. Moreover, elucidating the conformational changes in gM and its modulation of the fusion reaction is key. More importantly, to clear up a puzzle, which is the interaction of gM with other proteins how to affect cytokines and related signaling pathways, allows us to understand how the virus eludes the host immune system or suppresses the host immune response. A greater understanding of gM promotes the development of therapeutic inhibitors of fusion and entry.

## Author Contributions

All authors listed contributed to the completion of the article. CL and MW contributed to the design and writing of the article. RJ, QY, YW, DZ, XZ, SC, ML, SZ, XO, SM, QG, DS, XW, and BT all provided ideas contributing to this article’s conception and helped to create the table and figures. AC modified the article. All the authors reviewed and approved the final manuscript.

### Conflict of Interest

The authors declare that the research was conducted in the absence of any commercial or financial relationships that could be construed as a potential conflict of interest.
