# Supplementary material for: The Roles of Envelope Glycoprotein M in the Life Cycle of Some Alphaherpesviruses
Source: Front Microbiol. 2021 Feb 19;12:631523. doi: 10.3389/fmicb.2021.631523 (PMC7933518; doi:10.3389/fmicb.2021.631523)
Supplement: Supplementary file 1 [file Data_Sheet_1.pdf]

Supplementary Material

1 Supplementary Figures and Tables

For more information on Supplementary Material and for details on the different file types accepted, please see here. Figures, tables, and images will be published under a Creative Commons CC-BY licence and permission must be obtained for use of copyrighted material from other sources (including re-published/adapted/modified/partial figures and images from the internet). It is the responsibility of the authors to acquire the licenses, to follow any citation instructions requested by third-party rights holders, and cover any supplementary charges.

1.1 Supplementary Figures

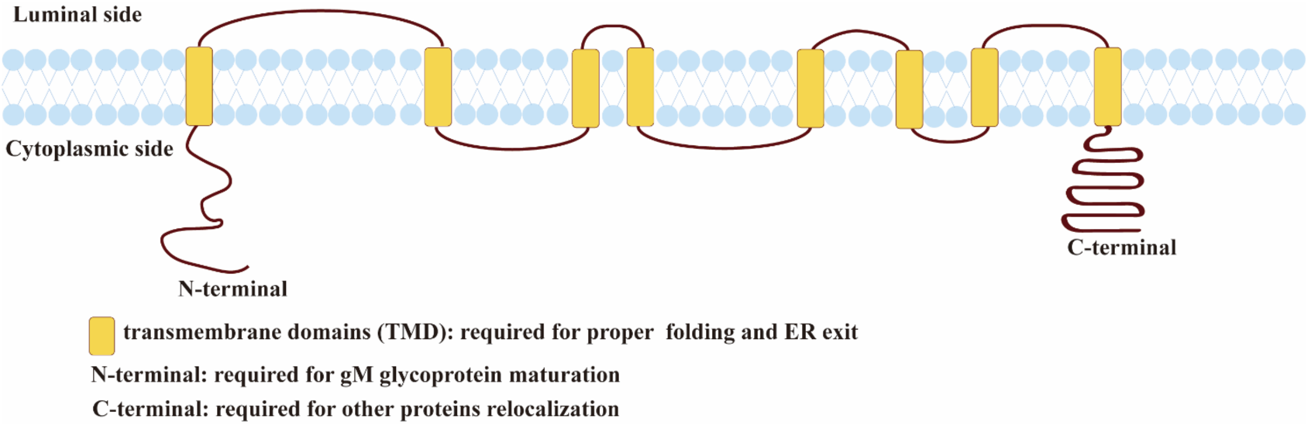

Supplementary Figure 1. Predicted topology of HSV-1 gM.

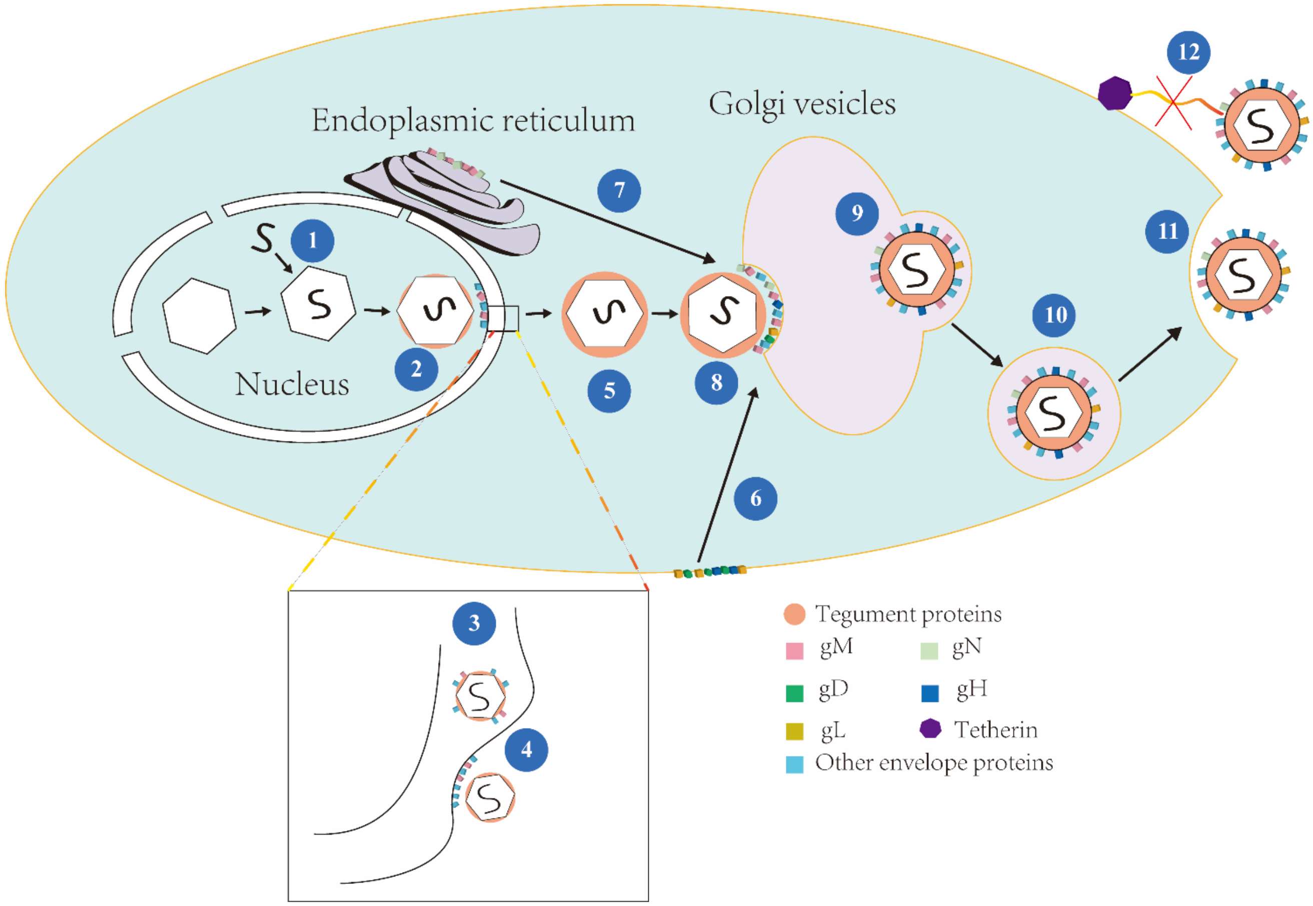

Supplementary Figure 2. The involvement of gM in the life cycle of HSV-1(Kelly et al., 2009).

1.2 Supplementary Tables

TABLE 1. gM gene of specific alphaherpesviruses and its function

Virus	Gene	Protein	Type of protein	Function in life cycle	references
HSV-1	UL10	gM	type III transmembrane protein	modulate the viral fusion machinery with gN	(Striebinger et al., 2015)
HSV-2	UL10	gM	/	/	(Dolan et al., 1998)
BHV-1	UL10	gM	type III transmembrane protein	Help gN and VP22 incorporate to virion	(Lipińska et al., 2006; Pannhorst et al., 2018)
EHV-1	ORF52	gM	type III transmembrane protein	involved in virus egress and fusion	(Rudolph and Osterrieder, 2002)
EHV-4	ORF52	gM	type III transmembrane protein	Ensure virus egress and cell-to-cell spread	(Ziegler et al., 2005)
VZV	ORF50	gM	type III transmembrane protein	Ensure virus cell-to-cell spread	(Yamagishi et al., 2008; Sadaoka et al., 2010; Lebrun et al., 2018)
PRV	UL10	gM	/	inhibits membrane fusion and involved in axonal sorting and transport	(Brack et al., 1999; Kratchmarov et al., 2015)
MDV	UL10	gM	/	essential for virus growth in cultured cells	(Cai et al., 1999; Tischer et al., 2002)
ILTV	UL10	gM	/	/	(Veits et al., 2003)
DEV	UL10	gM	/	/	(Wu et al., 2012)
